# The Hospital and the Surgeon

**Published:** 1916-11-04

**Authors:** S. S. Goldwater

**Affiliations:** Superintendent Mount Sinai Hospital, New York.


### THE HOSPITAL AND THE SURGEON.*

### I. THE SELECTION OF THE STAFF.

By S. S. GOLDWATER, M.D., Superintendent Mount Sinai Hospital, New York.

In the selection of its intern staff, the hospital to a great extent determines who shall and who shall not receive surgical training. The intern of to-day is the visiting surgeon and the surgical consultant of to-morrow; the selection and training of hospital interns is, therefore, of fundamental importance. It is certain that interns should not be assigned to their respective duties in the hospital in hit-or-miss fashion, but that their capacities, tendencies, and character should be carefully considered. After making the first departmental assignment, the hospital can do or leave undone much that is essential to proper surgical training.

The duration of the intern's service is important; a hospital may choose to give many men a smattering of surgical knowledge and a moderate degree of surgical skill, or it may train a few men thoroughly. The hospital which graduates a house surgeon in six months contributes little to surgical progress. At the other end of the scale is the hospital which, under a system of graduated promotions, keeps its residents indefinitely—a plan which has much to recommend it in individual instances, but the general adoption of which would unduly limit the privilege of surgical training. The middle course, which at present is followed by most hospitals in this country, provides for a period of training of not less than twelve and not more than twenty-four months.

### Dangers of Premature Specialisation.

No definite recommendation will here be made concerning the duration of surgical internship; I wish to lay stress on the quality of the training rather than its duration. For example, the intern whose aim is surgery should not have his attention directed to surgery exclusively at the outset of his hospital career; the training of the surgeon-to-be should preferably be "mixed," including a period of service in medical wards. The medical point of view, once acquired, will not be readily discarded; as an asset to the surgeon, it is invaluable.

So much time is necessarily spent by the surgical staff in performing operations and treating wounds, that the more refined methods of diagnosis and observation which are more easily and more generally practised in the medical wards are often neglected. The surgeon is frequently called on to treat a localised physical condition which is perfectly patent and which demands prompt operative relief; hence his characteristic habit of making a snap diagnosis, a method which, for surgical purposes, is often singularly effective. An abscess may advantageously be opened, or a hæmorrhage successfully controlled, without an exhaustive inquiry into the patient's family history, and without the performance of searching clinical tests. The habit of rapid diagnosis may, however, be carried too far. Certainly it tends to preclude the more careful, deliberate, and thorough investigation which is often essential to the correct diagnosis of obscure or complicated pathological conditions, and which is more likely to be practised by the visiting staff, and therefore learned by the intern, in the medical wards.

In ideal practice, a medical diagnostician would be associated with every busy surgeon. Under the ordinary conditions of private practice, such association is not always possible; the surgeon, consequently, must be not only operator, but diagnostician as well, and it is because, in after life, he will frequently carry this double responsibility, that the beginner should not be plunged too quickly into the alluring atmosphere of the operating-room, where all the conditions favour quick decision and action, but should first be trained thoroughly in diagnostic investigation, an art which attains its highest development in the medical wards.

If it is true that the surgeon should have some preliminary training in medical diagnosis and therapy, it is no less true that his preliminary education should include a course in pathology. Since we are viewing the subject from the point of view of hospital administration, let me suggest that the hospital laboratory be thrown open as widely as possible to the surgical intern and the junior visiting surgeon. The provision of opportunities for the study of pathology by those who are ultimately to be the hospital's chief surgeons will prove to be a profitable investment.

### Selection of Visiting Staff.

The selection of an attending surgeon is rarely accomplished without criticism. The criticism of disappointed candidates and their friends cannot be avoided, and the appointing authority need not take such criticism too seriously if the selection has been made conscientiously. Just how to ascertain the relative merits of the candidates is a difficult question.

In Chicago the municipal hospital authorities have adopted the plan of holding a competitive examination, and of appointing as visiting surgeon the candidate who obtains the highest rating. At first blush such a plan appears to be impracticable, but experience has demonstrated that it is possible to make surgical appointments or other professional appointments of similar grade under Civil Service rules, provided the rules are wisely framed. The

* We are indebted to the courtesy of Dr. Goldwater and the *Modern Hospital* for the copy of this paper, which was read before the American Hospital Conference, Philadelphia, September 1916.

rating of the candidates must, of course, be left to competent persons. One of the highest developments of the merit system is the employment by Civil Service Commissions of specially qualified examiners, expert in their respective fields.

A competitive examination, honestly conducted, will at least eliminate the totally unfit, a result which is by no means assured in municipal hospitals which are under unchecked partisan political control. It is just as practicable for Civil Service examiners as it is for private hospital boards to make proper allowance for personality, training, and for the diagnostic and operative skill shown in the past performance of the candidates; Civil Service examiners are under no compulsion to limit their attention to the candidate's ability to answer text-book questions.

Those who are disposed to regard the competitive system as impracticable are invited to compare the best results achieved under that system with the worst results obtained in municipalities where public and professional opinion has not been developed to the point of demanding a high quality of service in municipal hospitals, and where all checks on partisan abuse are absent. In the course of an official investigation of an important municipal hospital in 1913, the writer encountered an interesting case of stubborn rebellion on the part of the members of the house staff. The grievance of these men was that, after their appointment from the graduating class of the local medical school by means of a competitive examination, they found themselves suddenly confronted with a full-fledged visiting surgeon, formerly a member of their own class at medical school, *who had failed to qualify as an intern*, but whose political influence had been so potent as to accomplish his immediate elevation, with no preparation or fitness whatever, to the position of visiting surgeon. The indignation of this man's classmates was not without cause, nor their rebellion unjustified. To such a disgraceful and dangerous appointment the merit system presents an insuperable obstacle.

### Lay Elective Committees.

Lay trustees are probably more nearly impartial in the designation of medical appointees than medical men would be; laymen, at all events, are less likely to be influenced by personal or professional relations with the candidates, and are in a better position to make a calm and thorough canvass of the field in search of the best men available. Hospital trustees are not wholly unreasonable who decline to bestow the power of appointment on medical men without a check. They may wisely retain the appointing power in their own hands; but, recognising that technical matters are best judged by experts, they should not act without the assistance of medical advisers.

I have yet to encounter a safer system than that which provides that, on the occurrence of a staff vacancy, a committee jointly representing the lay board and the medical board be convened to prepare a list of men eligible to fill the vacant post. Any candidate who successfully withstands the fire of criticism which is levelled at him in the course of a joint committee meeting of this character is pretty certain to possess at least the necessary minimum qualifications. In one institution the list of eligibles created by the joint committee is subsequently entrusted to the lay committee alone; this committee makes a selection, and recommends the nominee to the medical and lay boards in turn for confirmation. This system includes all possible safeguards. Although the actual nomination is made by a committee of laymen, the proposal of any candidate whose qualifications have not been approved by the medical members of the joint committee is impossible. A further safeguard is provided by entrusting the veto power to two large bodies of medical men and laymen respectively.

But, in spite of all precautions, an appointment may prove to be unsatisfactory for one reason or another. A surgeon who is personally competent may fail to become a congenial or an efficient member of the staff. On account of this possibility, it is wise to regard all appointments in the junior grades, at least, as trial appointments only, subject to cancellation at the expiration of a probationary period. The idea that a surgeon who has once been appointed to a hospital position thereby acquires a lifelong claim on the job is inadmissible.

### Inappropriate Nominations.

The trustee's family physician is for ever bobbing up as a hospital candidate, seeking surgical or other important appointments. Within the past three years two of the most important medical administrative positions in the country, one a hospital superintendency and the other a health commissionership, have been given to the family physicians of mayors. In both instances these appointments were given to men wholly inexperienced in the work assigned to them, and claiming no suitable qualifications. It is with a view to the prevention of mistakes of this sort that it is suggested that hospital appointing committees include members of the medical staff; by this means the error of any trustee who might venture to make a wholly inappropriate nomination would be readily disclosed.

In the selection of a surgeon, trustees may be unduly impressed by popularity. A surgeon rarely acquires a large *clientèle* unless he is competent to operate, but the most popular surgeon is not always entitled to hospital preferment. A surgeon whose private practice is so large as to exhaust his time and energy cannot be expected to be punctual in attendance on the wards, or to be deliberate, careful, and thorough in examining ward patients. This is the type of surgeon who sees his patient for the first time in the operating-room, and who is content to leave diagnosis and other preliminaries in the hands of the house staff. Such a surgeon may be entirely willing to give his best service to the hospital, but he is not in a position to do so.

The function of the hospital is a dual one—first, to care for its patients, and, secondly, to con-

(*Continued on page* 88.)

### THE HOSPITAL AND THE SURGEON—(*Continued from p.* 86.)

tribute to the advancement of medicine. No hospital may rightly neglect either of these functions. A surgeon who is so preoccupied by private practice that he cannot interest himself in research work, cannot train his assistants, and cannot co-operate freely with the laboratory staff is one whom the hospital can afford to pass by.

The mistake is often made of appointing to a hospital position a surgeon who is already identified with several other institutions; this is analogous to the appointment of a man who is over-occupied with private practice. It is evident that when such an appointment is made somebody must suffer. If the new appointment is more attractive than the old, the appointee will give more attention to his new than to his old responsibilities, a result which raises the question of fair play. It is conceivable that one hospital may rank distinctly higher than another in the professional opportunities which it offers, and that the appointment to such a hospital of a man already holding a position elsewhere would be a deserved promotion. The ranking hospital, under such circumstances, has the right to offer an appointment to the man of its choice; but if the appointment is accepted, the situation must be frankly faced by the appointee, who should not attempt to monopolise the field or to undertake impossibilities.

Hospitals depend on the goodwill of the communities they serve. Local public opinion, therefore, is nearly always an important factor in determining the official acts of the hospital management. To offend local opinion is not good policy; and yet it may be necessary to do this in order to protect the hospital's higher interests. If, for example, an important position is open, and the man who is most conspicuously qualified to fill it happens to be a resident of another city, local pride will rebel against his appointment and will urge the nomination of some local practitioner. The hospital board which encounters such a situation is in a difficult position. If the non-resident candidate is appointed, the hospital will lose the goodwill of some of its friends; but this loss is temporary, and in the long run will be offset by the added reputation which the better qualified candidate will eventually bring to the hospital. Geographical limitations, then, should not be permitted to govern too strictly. It is gratifying to observe that hospital boards are more and more asserting their right to look anywhere for men who are qualified to raise the hospital to the highest efficiency.

